# Presence of SARS-CoV-2 RNA on Surfaces of Public Places and a Transportation System Located in a Densely Populated Urban Area in South America

**DOI:** 10.3390/v14010019

**Published:** 2021-12-23

**Authors:** Juan José Guadalupe, María I. Rojas, Gabriela Pozo, Maria P. Erazo-Garcia, Pamela Vega-Polo, Martín Terán-Velástegui, Forest Rohwer, María de Lourdes Torres

**Affiliations:** 1 Laboratorio de Biotecnología Vegetal, Universidad San Francisco de Quito (USFQ), Diego de Robles y Via Interoceanica s/n, Quito 170157, Ecuador; jguadalupe@usfq.edu.ec (J.J.G.); gpozo@usfq.edu.ec (G.P.); ma.paula_erazo@hotmail.com (M.P.E.-G.); pamelavegap3108@outlook.com (P.V.-P.); materanv96@gmail.com (M.T.-V.); 2 Biology Department, San Diego State University, San Diego, CA 92182, USA; mariaisabelrm@gmail.com (M.I.R.); frohwer@gmail.com (F.R.); 3 Viral Information Institute, San Diego State University, San Diego, CA 92182, USA

**Keywords:** COVID-19, coronavirus, environmental virus, SARS-CoV-2 RNA

## Abstract

Severe acute respiratory syndrome coronavirus 2 (SARS-CoV-2) is a highly transmissible RNA virus that causes COVID-19. Being aware of the presence of the virus on different types of surfaces and in different environments, and having a protocol for its detection, is important to understand the dynamics of the virus and its shedding patterns. In Ecuador, the detection of viral RNA in urban environmental samples has not been a priority. The present study analyzed samples from two densely populated neighborhoods and one public transportation system in Quito, Ecuador. Viral RNA presence was assessed using RT-LAMP. Twenty-eight out of 300 surfaces tested positive for SARS-CoV-2 RNA (9.33%). Frequently touched surfaces, especially in indoor spaces and on public transportation, were most likely to be positive for viral RNA. Positivity rate association for the two neighborhoods and for the surface type was not found. This study found viral RNA presence on urban surfaces; this information provides an insight into viral dissemination dynamics. Monitoring environmental SARS-CoV-2 could support the public health prevention strategies in Quito, Ecuador.

## 1. Introduction

Severe acute respiratory syndrome coronavirus 2 (SARS-CoV-2) is a highly transmissible, positive-sense, single-stranded RNA virus [1]. It is the vector pathogen of the coronavirus disease 2019 (COVID-19), an illness characterized by fever, cough, and fatigue, which can develop into severe cases of pneumonia [2]. COVID-19 emerged in Wuhan, China, in November 2019, and has spread rapidly throughout the world. In March 2020, the disease was officially declared a pandemic by the World Health Organization [1]. Since then, a growing number of SARS-CoV-2 infections have imposed unprecedented pressure on the economic and healthcare systems of both developed and developing countries [3].

In Ecuador, the first official case of COVID-19 was reported in late February 2020, and measures taken by the Ecuadorian government to contain the virus included home confinement with the suspension of all non-essential activities [4]. However, during the following months, COVID-19 positive cases were reported throughout the country. The virus spread rapidly, especially within the largest cities, such as Guayaquil and Quito, and led to one of the worst excess in mortality per million inhabitants rates in the region [5]. As of 15 November 2021, the number of cases in Ecuador has continued to increase, with 519,560 confirmed cases of COVID-19 [1].

The high transmissibility of SARS-CoV-2 is a key factor explaining the persistence of COVID-19. The primary route of viral transmission is direct person-to-person contact, through respiratory droplets. Additionally, alternative routes of transmission have been proposed via indirect contact through fomites and SARS-CoV-2 positive surfaces, as large amounts of SARS-CoV-2 can cause some virus to remain viable on dry surfaces and in aerosols for long periods of time [1,6,7,8].

In previous studies, the stability of SARS-CoV-2 has been assessed in different environmental conditions. In general, under typical indoor environmental conditions, the virus is expected to survive for up to 3 days on non-porous surfaces, such as stainless steel, plastic, and glass [9]. However, the virus has been shown to be more stable on plastic and stainless steel, and viable viral particles have been recovered from these surfaces up to 21 days and 28 days after their contact with the virus, respectively [10,11]. These noteworthy extensive periods of viral recovery on surfaces have been reported under laboratory conditions. Nevertheless, the high inoculum viral concentration inputs, the inability to show a correlation between the viral RNA levels and infectious virus, the use of optimal temperature and humidity conditions that are favorable for virus survival, and UV light protection have been pointed out as biases in these studies [12].

SARS-CoV-2 RNA detection in the environment could be an indicator of viral shedding [13]. Being aware of viral presence is important, as human–virus cohabitation can have an impact on public health [14]. The dynamics of viral circulation and community composition in urban environments are still largely unknown. In order to gain more insight into viral dynamics, we must first be able to detect its presence [15].

In Ecuador, SARS-CoV-2 research has mainly been focused on identifying the virus in patient samples and sequencing the genomes of the different variants in circulation [16,17,18]; however, viral presence on surfaces has not been studied and is unknown. Therefore, the present study aimed to analyze the presence of SARS-CoV-2 RNA on surfaces in two neighborhoods and a public transportation system in Quito, Ecuador, through multiplex reverse-transcription loop-mediated isothermal amplification (RT-LAMP).

## 2. Materials and Methods

### 2.1. Study Site

**Figure 1:**
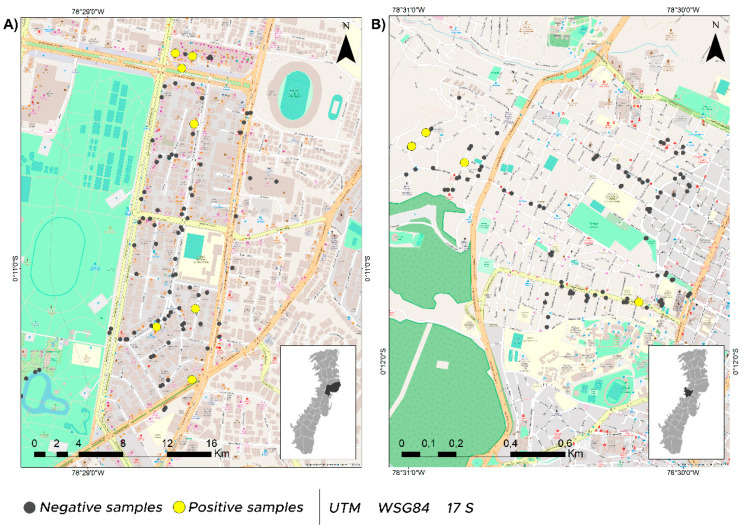
Sampling locations in Quito metropolitan district (DMQ), negative samples are represented with a grey dot and positive samples are shown with a yellow dot. (**A**) Iñaquito neighborhood sampling sites, (**B**) Belisario Quevedo neighborhood sampling sites. Map made with ArcGis 10.5.

A total of 300 environmental samples were collected in the city of Quito, Ecuador, during August 2020 in two neighborhoods (Belisario Quevedo and Iñaquito) and one public transportation service (Figure 1, Table S1). The two selected neighborhoods are characterized by being highly commercial and densely populated, with approximately 42,000 to 46,000 inhabitants according to the latest population census [19]. In August 2020, Belisario Quevedo was one of the neighborhoods with the highest prevalence of COVID-19 cases (~935) in Quito, while Iñaquito had fewer cases (~241) [20]. The public transportation system we sampled is one of the most important in the city, with hundreds of thousands of passengers per year [21]. Within this public transportation system, different bus units in movement were sampled. The sampling sites were categorized into the following five major groups: (1) parks, (2) shops/business premises, (3) restaurants/minimarkets, (4) transportation, and (5) other public places (OPP), which included benches, bus stops, doorbells, handles, among other highly touched surfaces. Sampled surface materials included wood, metal, plastic, glass and ceramic. The environment type (indoor or outdoor) was also taken into account when sampling. These groupings were used as parameters for the statistical analyses (Table S1).

### 2.2. Sampling and Detection

Sample collection was performed following the protocol of Rojas et al., 2021. Briefly, samples were collected using polyester swabs soaked in 0.5% SDS (sodium dodecyl sulfate) (HiMedia Laboratories, Chester, PA, USA), which inactivates and stabilizes the viral genetic material. An area of 10 × 10 cm was covered with a swab that was then placed in 1.5 mL tubes with 150 uL TRIzol reagent (Ambion, Carlsbad, CA, USA). Samples were stored at −80 °C for downstream processing. The type of surface material and type of environment (indoor/outdoor) for each sample were recorded. RNA isolation was performed using a TRIzol/chloroform-based protocol (Ambion, Carlsbad, CA, USA) (Pharmaco-Aaper, Brookfield, CT, USA). SARS-CoV-2 detection was performed using RT-LAMP according to Rojas et al., 2021. Colorimetric WarmStart^®^ LAMP Kit (NEB, Ipswich, MA, USA) was used with specific SARS-CoV-2 primers targeting the nucleocapsid (N2) and envelope (E1) genes. Amplification products were visualized in a 1.5% agarose gel (Figure S1).

### 2.3. Statistical Analyses

Chi-square and Fisher’s exact tests were used to analyze the positivity rates between neighborhoods, surface materials, environment types, and sampling sites depending on cell size. When entries included zeros or small numbers, chi-square test was used; otherwise, Fisher’s exact test was employed. Therefore, chi-square test was used for the categories ‘environment type’ and ‘neighborhood’, and Fisher’s exact test for ‘surface material’ and ‘sampling site’. Within the ‘sampling site’ category, chi-square test was used for comparisons between transportation and restaurants/minimarkets, shops/business premises and OPP, while Fisher’s exact test was used for all other comparisons. The strength of association between variables was measured by calculating Cramér’s V coefficient. Parameters were used as categorical independent variables in a logistic regression model to determine the effect of each variable on the presence/absence of SARS-CoV-2 RNA. Separate models were developed for each parameter and the association between variables was expressed as odds ratios (ORs) with the respective 95% confidence intervals (CIs). Bivariate analyses and modeling were performed using R programming software version 3.6.3 (R Foundation for Statistical Computing, Vienna, Austria).

## 3. Results

### 3.1. Viral SARS-CoV-2 RNA Presence

**Figure 2:**
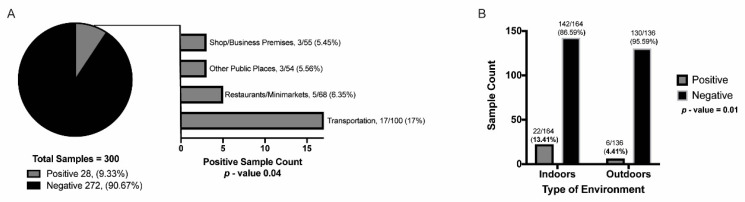
Percentage of positive and negative samples for SARS-CoV-2 viral RNA, and its distribution. (**A**) Percentage of positive samples found at the following different sampling sites: shop/business premises, other public places, restaurants/minimarkets, transportation (parks category not included as there were no positive samples found), and (**B**) percentage of positive and negative samples by type of environment (indoor/outdoor). The *p*-values obtained from the bivariate analyses are displayed.

The overall SARS-CoV-2 positivity rate was 9.33% (28/300 samples) (Figure 1). Highly touched public transport surfaces were the most likely to be SARS-CoV-2 positive, followed by restaurants/minimarkets, OPP, and shops/business premises (Figure 2A). No surface contamination with SARS-CoV-2 RNA was detected in parks. Positive samples were collected from the pen shaft of a minimarket counter, the elevator button and bathroom door handle of a shopping mall, the bench of a bus stop, two house doorbells, the counter of a stationery shop, and the table, chair, freezer and metal bars of restaurants or small food businesses. Regarding transport units, 13 metal and 4 plastic bus handles were positive for SARS-CoV-2 RNA. Of these, 22 samples were collected from indoor environments and 6 from outdoor environments (Figure 2B).

### 3.2. Positivity Rate Association between Parameters

We did not find a significant association between the positivity rate and the neighborhoods from which the samples were taken (*p*-value = 0.54), nor was there an association between the positivity rates found on different surface materials (*p*-value = 0.81). For the first parameter, seven positive samples were found in Iñaquito (7.00%) and four in Belisario Quevedo (4.00%). For the latter parameter, SARS-CoV-2 positive samples were collected from wood (2/18, 11.11%), metal (18/177, 10.17%), and plastic (8/84, 9.52%) surfaces. The samples collected from glass (0/17) and ceramic (0/4) surfaces were all negative. In contrast, we found a significant association between the positivity rate and the type of environment (*p*-value = 0.01, V = 0.15). In this case, the odds of a sample from an indoor environment being positive is 3.36 times higher than the odds of an outdoor environment sample being positive (*p*-value = 0.01) (Table 1). The positivity rate also showed a significant association with the sampling site (*p*-value = 0.04, V = 0.19), where the odds of a sample from the public transport units being positive is 3.31 times higher than the odds of a positive sample being obtained from an OPP (*p*-value = 0.04) (Table 1).

## 4. Discussion

We report a general positivity rate of 9% for our samples (Figure 2A), which is similar to previous reports that analyzed environmental surfaces (4.26–5.25%) [3,22]. Most of the research has focused on RNA detection in healthcare, and only a few studies have explored the presence of viral RNA in non-medical environments. Surface contamination has been explained by variables such as poor ventilation, frequency of touch, and variation in disinfection cleaning procedures [6].

In this study, transportation was the sampling site with the highest positivity rate (Figure 2A). SARS-CoV-2 RNA has also been found on surface swabs and in air samples inside public buses of densely populated cities, such as Barcelona and Tehran [7,23]. Other studies have reported viral RNA presence on food- and drink-related surfaces, and in public markets [3,24], which is a result that was also true for our study, where minimarkets and food-related premises had the second highest positivity rate. When testing swabs from shops/business premises and OPP, we detected viral RNA from samples taken from a bathroom door handle, doorbells, elevator buttons, a bus stop bench, and a service counter. Similarly, frequently touched surfaces, such as buttons (water fountains, automated teller machines, and elevators), counters, benches, and handrails on bus terminals, have been reported as positive for SARS-CoV-2 contamination in other studies [3,22,24,25,26]. In addition, bathroom door handles are surfaces that are commonly reported as SARS-CoV-2 positive, probably due to fecal-derived aerosols, which can be positive for SARS-CoV-2 [25,27]. For open public places, such as parks, we did not find SARS-CoV-2 RNA. Similarly, Abrahao et al. (2021) did not identify viral RNA in parks and on playground surfaces, and Kozer et al. (2021) only found RNA on 2 out of 43 sampled surfaces from playground equipment.

The discrepancies in the samples positive for SARS-CoV-2 RNA found in our study could be related to the type of environment and the inner characteristics of each sampling site. Bulfone et al. (2021) reported that the odds of indoor transmission are very high compared to outdoor transmission (18.7 times). In accordance, we found a significant association (*p*-value = 0.01) between the positivity rate and the type of environment, with a higher probability of obtaining positive samples indoors (3.36 times) (Table 1) [28]. Aerosolized droplets from an infected person can easily settle and persist on immediate surfaces for extended periods, especially in poorly ventilated indoor spaces with a constant affluence of people [2,6,22], explaining the higher percentage of positive samples found in indoor environments (Figure 2B). Indoor environments are generally characterized by conditions that promote viral particle survival, such as temperatures below 20 °C, artificial ventilation, and the absence of ventilation, all of which stabilize the humidity and temperature [29]. In contrast, the environmental conditions to which outdoor spaces are subjected, such as temperature and exposure to direct sunlight, are related to the rapid decay of the virus [2,22]. In fact, parameters such as temperature have an important influence on viral decay. SARS-CoV-2 is stable at 4 °C, but is sensitive to heat, and viral inactivation can occur within 5 min at 70 °C. Furthermore, UV light has also been shown to be effective against the virus; with a UV index of 10, 99.9% of the viral particles on a surface are inactivated after one hour. Due to its location and geography, Quito has high UV indexes—frequently greater than 11—which possibly explains the low number of viral particles found in outdoor locations in our sampling [6,30].

We did not find a significant association between the positivity rates for the two neighborhoods. This result was unexpected, as Belisario Quevedo was one of the neighborhoods with the highest prevalence of COVID-19 cases in Quito at the time this study was conducted; nevertheless, this could be explained by the fact that the entire city was under quarantine and strict vehicular circulation measures [31]. These measures were especially strict in Belisario Quevedo, such as military presence to enforce compliance [32]. Therefore, this could explain why the detection of SARS-CoV-2 RNA was similar in both neighborhoods.

There was no significant association between the positivity rates for different surface material types. Previous studies have suggested that the stability of SARS-CoV-2 varies according to surface type; however, with our results, we can only highlight that SARS-CoV-2 RNA was more commonly found on plastic and metal surfaces. It is important to note that these surfaces comprised most of the analyzed samples (metal = 177 samples, plastic = 84 samples); therefore, this could represent a bias when assessing the association. However, it is important to mention that previous studies assessing viral particle stability on surfaces have found that the virus is more stable on plastic and stainless steel. These studies have found viable particles, under controlled conditions, up to 21 to 28 days following the contact of the virus with the surface, respectively, which could explain our elevated positivity rates on these surfaces [10,11].

It is important to consider that the present study had some limitations. Firstly, the limit of detection for the technique we employed is 10 viral RNA copies [8]. Therefore, if there were less than 10 viral RNA copies on a given surface, the virus was not detected, resulting in a false negative. Furthermore, no conclusions can be reached regarding SARS-CoV-2 persistence over time, since the sampling was limited to two neighborhoods in Quito and one public transportation system, during a period of 1 day each. Finally, variables such as time, temperature, and humidity [2], as well as the changing home confinement measures applied in Quito, may alter the results found in this study.

Our study demonstrates the presence of SARS-CoV-2 RNA on the surfaces in public places and transportation systems located in Quito, Ecuador. We found that there is a higher probability of SARS-CoV-2 RNA being present on indoor surfaces (*p* < 0.05), especially on public transportation and on highly touched surfaces. Even though indirect viral transmission is now considered to be very low [12,33], the presence of contamination on surfaces could also work as an indicator of viral shedding from infected subjects, or ineffective cleaning and disinfection. Moreover, understanding the impact of viral presence in cities has proved to be relevant to public health [14,15,34]. The present study provides insights into viral dissemination dynamics in a highly populated city. Considering the present findings, we recommend expanding the sampling sites and assessing the viability of the virus to further monitor the presence of SARS-CoV-2 in the environment in Quito.

## Figures and Tables

**Table 1:** Results of the logistic regression models for the effects of different parameters on the presence of SARS-CoV-2 RNA in environmental samples. Independent variables “environment—indoor” and “sampling site—transportation” are significantly associated with positivity rates in our sampling.

Independent Variables	Levels *	Odds Ratio (95% CI)	*p*-Value
Neighborhood	Iñaquito	1.81 (0.53, 7.09)	0.36
			
Surface material	Metal	2.09 (0.57, 13.54)	0.34
	Plastic	1.95 (0.46, 13.33)	0.41
			
Environment	Indoor	3.36 (1.40, 9.35)	0.01 **
Sampling site	Parks	0.21 (−4.72, 1.49)	0.30
	Restaurants/Minimarkets	1.29 (−1.26, 1.58)	0.70
	Shops/Business Premises	0.96 (−1.47, 1.49)	0.95
	Transportation	3.31 (0.06, 2.29)	0.04 **

* Reference levels for each parameter are as follows: neighborhood (Belisario Quevedo), surface material (others), environment (outdoor), sampling site (other public places). ** represents parameters that are significantly associated with positivity rates in our sampling.

## Data Availability

The data presented in this study are available in Table S1.

## Supplementary Materials

The following is available online at https://www.mdpi.com/article/10.3390/v14010019/s1: Table S1: metadata for all samples, including neighborhood, surface material, environment, sampling site and date, and SARS-CoV-2 RNA detection; Figure S1: detection of ladder pattern post LAMP assay for identifying SARS-CoV-2 RNA in environmental samples.
