# Supplementary material for: Presence of SARS-CoV-2 RNA on Surfaces of Public Places and a Transportation System Located in a Densely Populated Urban Area in South America
Source: Viruses. 2021 Dec 23;14(1):19. doi: 10.3390/v14010019 (PMC8780916; doi:10.3390/v14010019)
Supplement: Supplementary file 1 [file viruses-14-00019-s001.zip › viruses-1498449-supplementary.pdf]

Table S1: Metadata for all samples, including neighborhood, surface material, environment, sampling site and date, and SARS-CoV-2 RNA detection

Sample	Neighborhood	Surface material	Environment	Sampling site	Sampling Date	SARS-CoV-2 RNA detection
1	Iñaquito	Plastic	Indoor	Restaurants	08/10/2020	Positive
2	Iñaquito	Glass	Indoor	Shops	08/10/2020	Negative
3	Iñaquito	Metal	Outdoor	Restaurants	08/10/2020	Negative
4	Iñaquito	Plastic	Outdoor	Restaurants	08/10/2020	Negative
5	Iñaquito	Wood	Indoor	Restaurants	08/10/2020	Negative
6	Iñaquito	Metal	Outdoor	Restaurants	08/10/2020	Negative
7	Iñaquito	Plastic	Indoor	Restaurants	08/10/2020	Negative
8	Iñaquito	Metal	Indoor	Restaurants	08/10/2020	Negative
9	Iñaquito	Metal	Outdoor	Shops	08/10/2020	Negative
10	Iñaquito	Plastic	Outdoor	Restaurants	08/10/2020	Negative
11	Iñaquito	Plastic	Outdoor	Restaurants	08/10/2020	Negative
12	Iñaquito	Wood	Indoor	Shops	08/10/2020	Negative
13	Iñaquito	Plastic	Indoor	Restaurants	08/10/2020	Negative
14	Iñaquito	Metal	Indoor	Shops	08/10/2020	Negative
15	Iñaquito	Glass	Indoor	Restaurants	08/10/2020	Negative
16	Iñaquito	Metal	Indoor	Shops	08/10/2020	Negative
17	Iñaquito	Metal	Indoor	Shops	08/10/2020	Positive
18	Iñaquito	Metal	Indoor	Shops	08/10/2020	Negative
19	Iñaquito	Metal	Indoor	OPP	08/10/2020	Positive
20	Iñaquito	Metal	Outdoor	OPP	08/10/2020	Positive
21	Iñaquito	Plastic	Indoor	Shops	08/10/2020	Negative
22	Iñaquito	Plastic	Outdoor	Shops	08/10/2020	Negative
23	Iñaquito	Wood	Outdoor	Shops	08/10/2020	Positive
24	Iñaquito	Plastic	Outdoor	Shops	08/10/2020	Negative
25	Iñaquito	Plastic	Outdoor	OPP	08/10/2020	Negative
26	Iñaquito	Metal	Outdoor	OPP	08/10/2020	Negative
27	Iñaquito	Rock	Indoor	Restaurants	08/10/2020	Negative
28	Iñaquito	Plastic	Outdoor	OPP	08/10/2020	Negative
29	Iñaquito	Plastic	Indoor	OPP	08/10/2020	Negative
30	Iñaquito	Metal	Outdoor	OPP	08/10/2020	Negative
31	Iñaquito	Metal	Outdoor	OPP	08/10/2020	Negative
32	Iñaquito	Wood	Outdoor	Shops	08/10/2020	Negative
33	Iñaquito	Plastic	Indoor	OPP	08/10/2020	Negative
34	Iñaquito	Metal	Outdoor	OPP	08/10/2020	Negative
35	Iñaquito	Metal	Outdoor	OPP	08/10/2020	Negative
36	Iñaquito	Wood	Indoor	Parks	08/10/2020	Negative
37	Iñaquito	Glass	Outdoor	Restaurants	08/10/2020	Negative
38	Iñaquito	Metal	Outdoor	OPP	08/10/2020	Positive
39	Iñaquito	Glass	Outdoor	OPP	08/10/2020	Negative
40	Iñaquito	Plastic	Outdoor	OPP	08/10/2020	Negative
41	Iñaquito	Wood	Outdoor	Restaurants	08/10/2020	Negative
42	Iñaquito	Plastic	Outdoor	OPP	08/10/2020	Negative
43	Iñaquito	Glass	Outdoor	Restaurants	08/10/2020	Negative
44	Iñaquito	Metal	Outdoor	OPP	08/10/2020	Negative

45	Iñaquito	Plastic	Outdoor	OPP	08/10/2020	Negative
46	Iñaquito	Wood	Outdoor	Restaurants	08/10/2020	Negative
47	Iñaquito	Metal	Outdoor	OPP	08/10/2020	Negative
48	Iñaquito	Metal	Outdoor	OPP	08/10/2020	Negative
49	Iñaquito	Metal	Outdoor	OPP	08/10/2020	Negative
50	Iñaquito	Metal	Outdoor	OPP	08/10/2020	Negative
51	Iñaquito	Metal	Outdoor	OPP	08/10/2020	Negative
52	Iñaquito	Metal	Outdoor	Parks	08/10/2020	Negative
53	Iñaquito	Metal	Outdoor	Parks	08/10/2020	Negative
54	Iñaquito	Metal	Outdoor	Parks	08/10/2020	Negative
55	Iñaquito	Metal	Outdoor	Parks	08/10/2020	Negative
56	Iñaquito	Metal	Outdoor	Parks	08/10/2020	Negative
57	Iñaquito	Metal	Outdoor	Parks	08/10/2020	Negative
58	Iñaquito	Metal	Outdoor	Parks	08/10/2020	Negative
59	Iñaquito	Metal	Outdoor	Parks	08/10/2020	Negative
60	Iñaquito	Plastic	Outdoor	OPP	08/10/2020	Negative
61	Iñaquito	Wood	Indoor	Restaurants	08/10/2020	Negative
62	Iñaquito	Metal	Outdoor	Parks	08/10/2020	Negative
63	Iñaquito	Metal	Outdoor	Parks	08/10/2020	Negative
64	Iñaquito	Metal	Outdoor	Parks	08/10/2020	Negative
65	Iñaquito	Plastic	Outdoor	OPP	08/10/2020	Negative
66	Iñaquito	Metal	Indoor	Restaurants	08/10/2020	Negative
67	Iñaquito	Plastic	Outdoor	OPP	08/10/2020	Negative
68	Iñaquito	Plastic	Indoor	OPP	08/10/2020	Negative
69	Iñaquito	Metal	Outdoor	Restaurants	08/10/2020	Negative
70	Iñaquito	Metal	Outdoor	Restaurants	08/10/2020	Negative
71	Iñaquito	Plastic	Outdoor	OPP	08/10/2020	Negative
72	Iñaquito	Metal	Outdoor	Restaurants	08/10/2020	Negative
73	Iñaquito	Plastic	Outdoor	OPP	08/10/2020	Negative
74	Iñaquito	Plastic	Indoor	OPP	08/10/2020	Negative
75	Iñaquito	Metal	Outdoor	Restaurants	08/10/2020	Negative
76	Iñaquito	Metal	Outdoor	Restaurants	08/10/2020	Negative
77	Iñaquito	Metal	Outdoor	Restaurants	08/10/2020	Negative
78	Iñaquito	Metal	Outdoor	Restaurants	08/10/2020	Negative
79	Iñaquito	Metal	Outdoor	Restaurants	08/10/2020	Negative
80	Iñaquito	Glass	Indoor	Restaurants	08/10/2020	Negative
81	Iñaquito	Wood	Outdoor	Shops	08/10/2020	Negative
82	Iñaquito	Metal	Outdoor	Shops	08/10/2020	Negative
83	Iñaquito	Metal	Outdoor	Shops	08/10/2020	Negative
84	Iñaquito	Plastic	Outdoor	OPP	08/10/2020	Negative
85	Iñaquito	Metal	Outdoor	Shops	08/10/2020	Negative
86	Iñaquito	Plastic	Outdoor	OPP	08/10/2020	Negative
87	Iñaquito	Plastic	Outdoor	OPP	08/10/2020	Positive
88	Iñaquito	Metal	Outdoor	Shops	08/10/2020	Negative
89	Iñaquito	Metal	Outdoor	Shops	08/10/2020	Negative
90	Iñaquito	Plastic	Outdoor	Restaurants	08/10/2020	Negative
91	Iñaquito	Metal	Outdoor	Shops	08/10/2020	Negative
92	Iñaquito	Metal	Outdoor	Shops	08/10/2020	Negative
93	Iñaquito	Plastic	Indoor	Shops	08/10/2020	Negative

94	Iñaquito	Metal	Indoor	Shops	08/10/2020	Negative
95	Iñaquito	Metal	Outdoor	Shops	08/10/2020	Negative
96	Iñaquito	Metal	Outdoor	Shops	08/10/2020	Negative
97	Iñaquito	Metal	Outdoor	Shops	08/10/2020	Negative
98	Iñaquito	Metal	Outdoor	Shops	08/10/2020	Negative
99	Iñaquito	Metal	Outdoor	Shops	08/10/2020	Negative
100	Iñaquito	Glass	Outdoor	Restaurants	08/10/2020	Negative
101	Belisario Quevedo	Metal	Indoor	Restaurants	08/11/2020	Negative
102	Belisario Quevedo	Wood	Indoor	Restaurants	08/11/2020	Positive
103	Belisario Quevedo	Metal	Outdoor	Restaurants	08/11/2020	Negative
104	Belisario Quevedo	Metal	Outdoor	Restaurants	08/11/2020	Negative
105	Belisario Quevedo	Metal	Outdoor	Restaurants	08/11/2020	Negative
106	Belisario Quevedo	Metal	Outdoor	Restaurants	08/11/2020	Positive
107	Belisario Quevedo	Plastic	Outdoor	Restaurants	08/11/2020	Negative
108	Belisario Quevedo	Plastic	Outdoor	Restaurants	08/11/2020	Negative
109	Belisario Quevedo	Metal	Indoor	Restaurants	08/11/2020	Negative
110	Belisario Quevedo	Metal	Indoor	Restaurants	08/11/2020	Negative
111	Belisario Quevedo	Plastic	Outdoor	Restaurants	08/11/2020	Negative
112	Belisario Quevedo	Glass	Indoor	Restaurants	08/11/2020	Negative
113	Belisario Quevedo	Glass	Indoor	Restaurants	08/11/2020	Negative
114	Belisario Quevedo	Plastic	Indoor	Restaurants	08/11/2020	Negative
115	Belisario Quevedo	Metal	Indoor	Restaurants	08/11/2020	Negative
116	Belisario Quevedo	Plastic	Outdoor	Restaurants	08/11/2020	Negative
117	Belisario Quevedo	Metal	Outdoor	Restaurants	08/11/2020	Negative
118	Belisario Quevedo	Metal	Outdoor	Restaurants	08/11/2020	Negative
119	Belisario Quevedo	Plastic	Outdoor	Shops	08/11/2020	Positive
120	Belisario Quevedo	Wood	Outdoor	Restaurants	08/11/2020	Negative
121	Belisario Quevedo	Glass	Indoor	Restaurants	08/11/2020	Negative
122	Belisario Quevedo	Metal	Outdoor	Restaurants	08/11/2020	Negative
123	Belisario Quevedo	Plastic	Outdoor	OPP	08/11/2020	Negative
124	Belisario Quevedo	Plastic	Outdoor	OPP	08/11/2020	Negative
125	Belisario Quevedo	Metal	Outdoor	Restaurants	08/11/2020	Negative
126	Belisario Quevedo	Glass	Indoor	Shops	08/11/2020	Negative
127	Belisario Quevedo	Metal	Indoor	Shops	08/11/2020	Negative
128	Belisario Quevedo	Glass	Indoor	Shops	08/11/2020	Negative
129	Belisario Quevedo	Metal	Indoor	Shops	08/11/2020	Negative
130	Belisario Quevedo	Glass	Indoor	Shops	08/11/2020	Negative
131	Belisario Quevedo	Metal	Outdoor	OPP	08/11/2020	Negative
132	Belisario Quevedo	Rock	Outdoor	Restaurants	08/11/2020	Negative
133	Belisario Quevedo	Metal	Outdoor	OPP	08/11/2020	Negative
134	Belisario Quevedo	Plastic	Outdoor	OPP	08/11/2020	Negative
135	Belisario Quevedo	Glass	Indoor	Shops	08/11/2020	Negative
136	Belisario Quevedo	Metal	Outdoor	OPP	08/11/2020	Negative
137	Belisario Quevedo	Plastic	Outdoor	OPP	08/11/2020	Negative
138	Belisario Quevedo	Metal	Outdoor	OPP	08/11/2020	Negative
139	Belisario Quevedo	Metal	Indoor	OPP	08/11/2020	Negative
140	Belisario Quevedo	Metal	Indoor	OPP	08/11/2020	Negative
141	Belisario Quevedo	Wood	Indoor	Restaurants	08/11/2020	Negative
142	Belisario Quevedo	Wood	Indoor	Restaurants	08/11/2020	Negative

143	Belisario Quevedo	Plastic	Outdoor	OPP	08/11/2020	Negative
144	Belisario Quevedo	Metal	Outdoor	OPP	08/11/2020	Negative
145	Belisario Quevedo	Wood	Indoor	Shops	08/11/2020	Negative
146	Belisario Quevedo	Metal	Indoor	OPP	08/11/2020	Negative
147	Belisario Quevedo	Metal	Outdoor	OPP	08/11/2020	Negative
148	Belisario Quevedo	Wood	Indoor	Shops	08/11/2020	Negative
149	Belisario Quevedo	Metal	Outdoor	OPP	08/11/2020	Negative
150	Belisario Quevedo	Wood	Indoor	Shops	08/11/2020	Negative
151	Belisario Quevedo	Glass	Indoor	Shops	08/11/2020	Negative
152	Belisario Quevedo	Plastic	Indoor	OPP	08/11/2020	Positive
153	Belisario Quevedo	Metal	Outdoor	OPP	08/11/2020	Negative
154	Belisario Quevedo	Metal	Outdoor	OPP	08/11/2020	Negative
155	Belisario Quevedo	Plastic	Indoor	OPP	08/11/2020	Negative
156	Belisario Quevedo	Metal	Outdoor	OPP	08/11/2020	Negative
157	Belisario Quevedo	Plastic	Indoor	Parks	08/11/2020	Negative
158	Belisario Quevedo	Metal	Indoor	OPP	08/11/2020	Negative
159	Belisario Quevedo	Metal	Outdoor	OPP	08/11/2020	Negative
160	Belisario Quevedo	Rock	Outdoor	Parks	08/11/2020	Negative
161	Belisario Quevedo	Rock	Indoor	Parks	08/11/2020	Negative
162	Belisario Quevedo	Metal	Outdoor	Parks	08/11/2020	Negative
163	Belisario Quevedo	Metal	Outdoor	Parks	08/11/2020	Negative
164	Belisario Quevedo	Metal	Outdoor	Parks	08/11/2020	Negative
165	Belisario Quevedo	Metal	Outdoor	Parks	08/11/2020	Negative
166	Belisario Quevedo	Metal	Outdoor	Parks	08/11/2020	Negative
167	Belisario Quevedo	Metal	Outdoor	Parks	08/11/2020	Negative
168	Belisario Quevedo	Metal	Outdoor	Parks	08/11/2020	Negative
169	Belisario Quevedo	Metal	Outdoor	Parks	08/11/2020	Negative
170	Belisario Quevedo	Metal	Outdoor	Restaurants	08/11/2020	Negative
171	Belisario Quevedo	Plastic	Outdoor	Restaurants	08/11/2020	Negative
172	Belisario Quevedo	Wood	Indoor	Shops	08/11/2020	Negative
173	Belisario Quevedo	Metal	Outdoor	Restaurants	08/11/2020	Negative
174	Belisario Quevedo	Plastic	Outdoor	Restaurants	08/11/2020	Negative
175	Belisario Quevedo	Glass	Indoor	Shops	08/11/2020	Negative
176	Belisario Quevedo	Plastic	Outdoor	Restaurants	08/11/2020	Negative
177	Belisario Quevedo	Metal	Outdoor	Restaurants	08/11/2020	Negative
178	Belisario Quevedo	Plastic	Outdoor	Restaurants	08/11/2020	Negative
179	Belisario Quevedo	Metal	Outdoor	Restaurants	08/11/2020	Negative
180	Belisario Quevedo	Metal	Outdoor	Restaurants	08/11/2020	Negative
181	Belisario Quevedo	Metal	Outdoor	Restaurants	08/11/2020	Negative
182	Belisario Quevedo	Metal	Outdoor	Restaurants	08/11/2020	Negative
183	Belisario Quevedo	Metal	Outdoor	Restaurants	08/11/2020	Negative
184	Belisario Quevedo	Metal	Indoor	Restaurants	08/11/2020	Negative
185	Belisario Quevedo	Metal	Outdoor	Shops	08/11/2020	Negative
186	Belisario Quevedo	Plastic	Indoor	Shops	08/11/2020	Negative
187	Belisario Quevedo	Wood	Indoor	Restaurants	08/11/2020	Negative
188	Belisario Quevedo	Metal	Outdoor	Shops	08/11/2020	Negative
189	Belisario Quevedo	Metal	Outdoor	Shops	08/11/2020	Negative
190	Belisario Quevedo	Plastic	Indoor	Shops	08/11/2020	Negative
191	Belisario Quevedo	Plastic	Outdoor	Shops	08/11/2020	Negative

192	Belisario Quevedo	Metal	Outdoor	Shops	08/11/2020	Negative
193	Belisario Quevedo	Metal	Outdoor	Shops	08/11/2020	Negative
194	Belisario Quevedo	Metal	Indoor	Shops	08/11/2020	Negative
195	Belisario Quevedo	Plastic	Outdoor	Shops	08/11/2020	Negative
196	Belisario Quevedo	Metal	Outdoor	Shops	08/11/2020	Negative
197	Belisario Quevedo	Plastic	Outdoor	Shops	08/11/2020	Negative
198	Belisario Quevedo	Metal	Indoor	Shops	08/11/2020	Negative
199	Belisario Quevedo	Glass	Indoor	Shops	08/11/2020	Negative
200	Belisario Quevedo	Metal	Indoor	Shops	08/11/2020	Negative
201	Transportation S.	Metal	Indoor	Transportation	08/12/2020	Negative
202	Transportation S.	Plastic	Indoor	Transportation	08/12/2020	Negative
203	Transportation S.	Metal	Indoor	Transportation	08/12/2020	Negative
204	Transportation S.	Metal	Indoor	Transportation	08/12/2020	Negative
205	Transportation S.	Plastic	Indoor	Transportation	08/12/2020	Negative
206	Transportation S.	Metal	Indoor	Transportation	08/12/2020	Negative
207	Transportation S.	Metal	Indoor	Transportation	08/12/2020	Negative
208	Transportation S.	Plastic	Indoor	Transportation	08/12/2020	Negative
209	Transportation S.	Metal	Indoor	Transportation	08/12/2020	Negative
210	Transportation S.	Metal	Indoor	Transportation	08/12/2020	Negative
211	Transportation S.	Plastic	Indoor	Transportation	08/12/2020	Negative
212	Transportation S.	Metal	Indoor	Transportation	08/12/2020	Negative
213	Transportation S.	Metal	Indoor	Transportation	08/12/2020	Negative
214	Transportation S.	Plastic	Indoor	Transportation	08/12/2020	Negative
215	Transportation S.	Metal	Indoor	Transportation	08/12/2020	Negative
216	Transportation S.	Metal	Indoor	Transportation	08/12/2020	Negative
217	Transportation S.	Plastic	Indoor	Transportation	08/12/2020	Negative
218	Transportation S.	Metal	Indoor	Transportation	08/12/2020	Negative
219	Transportation S.	Metal	Indoor	Transportation	08/12/2020	Negative
220	Transportation S.	Plastic	Indoor	Transportation	08/12/2020	Negative
221	Transportation S.	Metal	Indoor	Transportation	08/12/2020	Negative
222	Transportation S.	Metal	Indoor	Transportation	08/12/2020	Negative
223	Transportation S.	Plastic	Indoor	Transportation	08/12/2020	Negative
224	Transportation S.	Metal	Indoor	Transportation	08/12/2020	Negative
225	Transportation S.	Metal	Indoor	Transportation	08/12/2020	Negative
226	Transportation S.	Plastic	Indoor	Transportation	08/12/2020	Positive
227	Transportation S.	Metal	Indoor	Transportation	08/12/2020	Negative
228	Transportation S.	Metal	Indoor	Transportation	08/12/2020	Negative
229	Transportation S.	Plastic	Indoor	Transportation	08/12/2020	Negative
230	Transportation S.	Metal	Indoor	Transportation	08/12/2020	Negative
231	Transportation S.	Metal	Indoor	Transportation	08/12/2020	Negative
232	Transportation S.	Plastic	Indoor	Transportation	08/12/2020	Negative
233	Transportation S.	Metal	Indoor	Transportation	08/12/2020	Negative
234	Transportation S.	Metal	Indoor	Transportation	08/12/2020	Negative
235	Transportation S.	Plastic	Indoor	Transportation	08/12/2020	Negative
236	Transportation S.	Metal	Indoor	Transportation	08/12/2020	Negative
237	Transportation S.	Metal	Indoor	Transportation	08/12/2020	Negative
238	Transportation S.	Plastic	Indoor	Transportation	08/12/2020	Negative
239	Transportation S.	Metal	Indoor	Transportation	08/12/2020	Negative
240	Transportation S.	Metal	Indoor	Transportation	08/12/2020	Negative

241	Transportation S.	Plastic	Indoor	Transportation	08/12/2020	Negative
242	Transportation S.	Metal	Indoor	Transportation	08/12/2020	Negative
243	Transportation S.	Metal	Indoor	Transportation	08/12/2020	Positive
244	Transportation S.	Plastic	Indoor	Transportation	08/12/2020	Negative
245	Transportation S.	Metal	Indoor	Transportation	08/12/2020	Negative
246	Transportation S.	Metal	Indoor	Transportation	08/12/2020	Negative
247	Transportation S.	Plastic	Indoor	Transportation	08/12/2020	Negative
248	Transportation S.	Metal	Indoor	Transportation	08/12/2020	Positive
249	Transportation S.	Metal	Indoor	Transportation	08/12/2020	Positive
250	Transportation S.	Plastic	Indoor	Transportation	08/12/2020	Negative
251	Transportation S.	Metal	Indoor	Transportation	08/12/2020	Positive
252	Transportation S.	Metal	Indoor	Transportation	08/12/2020	Positive
253	Transportation S.	Plastic	Indoor	Transportation	08/12/2020	Negative
254	Transportation S.	Metal	Indoor	Transportation	08/12/2020	Positive
255	Transportation S.	Metal	Indoor	Transportation	08/12/2020	Positive
256	Transportation S.	Plastic	Indoor	Transportation	08/12/2020	Negative
257	Transportation S.	Metal	Indoor	Transportation	08/12/2020	Negative
258	Transportation S.	Metal	Indoor	Transportation	08/12/2020	Positive
259	Transportation S.	Plastic	Indoor	Transportation	08/12/2020	Positive
260	Transportation S.	Metal	Indoor	Transportation	08/12/2020	Positive
261	Transportation S.	Metal	Indoor	Transportation	08/12/2020	Positive
262	Transportation S.	Plastic	Indoor	Transportation	08/12/2020	Negative
263	Transportation S.	Metal	Indoor	Transportation	08/12/2020	Negative
264	Transportation S.	Metal	Indoor	Transportation	08/12/2020	Negative
265	Transportation S.	Plastic	Indoor	Transportation	08/12/2020	Positive
266	Transportation S.	Metal	Indoor	Transportation	08/12/2020	Negative
267	Transportation S.	Metal	Indoor	Transportation	08/12/2020	Positive
268	Transportation S.	Plastic	Indoor	Transportation	08/12/2020	Negative
269	Transportation S.	Metal	Indoor	Transportation	08/12/2020	Negative
270	Transportation S.	Metal	Indoor	Transportation	08/12/2020	Negative
271	Transportation S.	Plastic	Indoor	Transportation	08/12/2020	Negative
272	Transportation S.	Metal	Indoor	Transportation	08/12/2020	Positive
273	Transportation S.	Metal	Indoor	Transportation	08/12/2020	Negative
274	Transportation S.	Plastic	Indoor	Transportation	08/12/2020	Negative
275	Transportation S.	Metal	Indoor	Transportation	08/12/2020	Negative
276	Transportation S.	Metal	Indoor	Transportation	08/12/2020	Negative
277	Transportation S.	Plastic	Indoor	Transportation	08/12/2020	Negative
278	Transportation S.	Metal	Indoor	Transportation	08/12/2020	Negative
279	Transportation S.	Metal	Indoor	Transportation	08/12/2020	Negative
280	Transportation S.	Plastic	Indoor	Transportation	08/12/2020	Negative
281	Transportation S.	Metal	Indoor	Transportation	08/12/2020	Negative
282	Transportation S.	Metal	Indoor	Transportation	08/12/2020	Negative
283	Transportation S.	Plastic	Indoor	Transportation	08/12/2020	Negative
284	Transportation S.	Metal	Indoor	Transportation	08/12/2020	Negative
285	Transportation S.	Metal	Indoor	Transportation	08/12/2020	Negative
286	Transportation S.	Plastic	Indoor	Transportation	08/12/2020	Negative
287	Transportation S.	Metal	Indoor	Transportation	08/12/2020	Negative
288	Transportation S.	Metal	Indoor	Transportation	08/12/2020	Positive
289	Transportation S.	Plastic	Indoor	Transportation	08/12/2020	Positive

290	Transportation S.	Metal	Indoor	Transportation	08/12/2020	Negative
291	Transportation S.	Metal	Indoor	Transportation	08/12/2020	Negative
292	Transportation S.	Plastic	Indoor	Transportation	08/12/2020	Negative
293	Transportation S.	Metal	Indoor	Transportation	08/12/2020	Negative
294	Transportation S.	Metal	Indoor	Transportation	08/12/2020	Negative
295	Transportation S.	Plastic	Indoor	Transportation	08/12/2020	Negative
296	Transportation S.	Metal	Indoor	Transportation	08/12/2020	Negative
297	Transportation S.	Metal	Indoor	Transportation	08/12/2020	Negative
298	Transportation S.	Plastic	Indoor	Transportation	08/12/2020	Negative
299	Transportation S.	Metal	Indoor	Transportation	08/12/2020	Negative
300	Transportation S.	Metal	Indoor	Transportation	08/12/2020	Negative

OPP = Other Public Places

Transportation S.= Transportation System

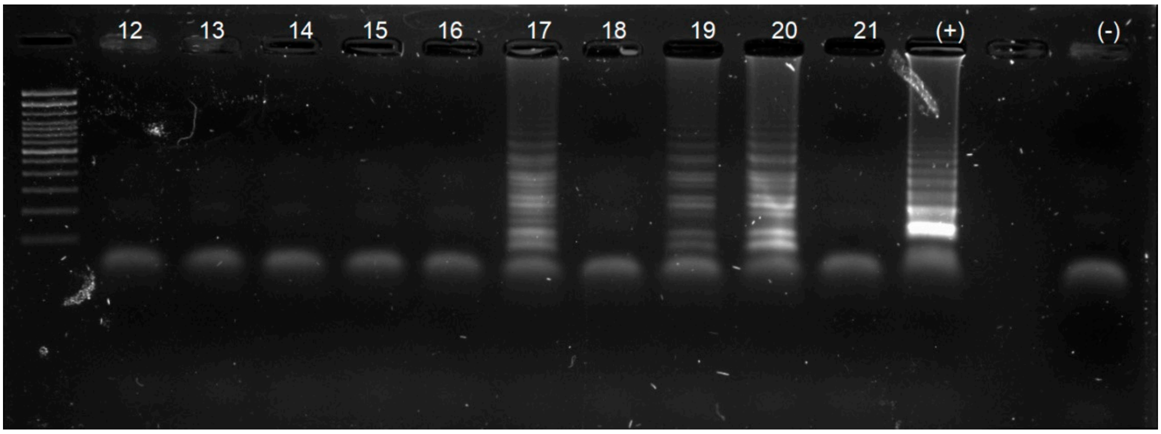

Figure S1: Detection of ladder pattern post LAMP assay for identifying SARS-CoV-2 RNA in environmental samples. Lane 1: 100 basepair ladder, Lanes 2- 6: samples 12-16 (negative), Lane 7: sample 17 (positive), Lane 8: sample 18 (negative), Lanes 9-10: samples 19-20 (positive), Lane 11: sample 21 (negative), Lane 12: positive control, Lane 13: empty, Lane 14: negative control.
